# Editorial: Bridging traditional knowledge and modern biomedical innovation (reviews in ethnopharmacology: 2025)

**DOI:** 10.3389/fphar.2026.1904841

**Published:** 2026-08-04

**Authors:** Fabien Schultz, Daniela Hanganu, Irina Ielciu

**Affiliations:** 1 Ethnopharmacology and Zoopharmacognosy, Bernhard Nocht Institute for Tropical Medicine, Hamburg, Germany; 2 Department of Agriculture and Food Sciences, Neubrandenburg University of Applied Sciences, Neubrandenburg, Germany; 3 Department of Pharmacognosy, Faculty of Pharmacy, “Iuliu Hatieganu” University of Medicine and Pharmacy Cluj-Napoca, Cluj-Napoca, Romania; 4 Department of Pharmaceutical Botany, Faculty of Pharmacy, “Iuliu Hatieganu” University of Medicine and Pharmacy Cluj-Napoca, Cluj-Napoca, Romania

**Keywords:** ethnopharmacology, herbal drugs, medicinal plants, phytomedicine, traditional medicine

The special issue “Reviews in Ethnopharmacology: 2025 - Bridging Traditional Knowledge and Modern Biomedical Innovation,” published in *Frontiers in Pharmacology,* represents a milestone in the continuing evolution of ethnopharmacological research. Bringing together 48 publications from an international network of scholars, clinicians, pharmacologists, botanists, and translational researchers, this collection highlights the growing global relevance of traditional medicine and natural-product research in addressing contemporary healthcare challenges. A total of 328 authors have contributed to this Research Topic that has garnered significant global attention, with more than 221,000 topic views, 170,000 article views, and 44,000 article downloads, as on 26 May 2026.

Published within the Ethnopharmacology section, the Research Topic demonstrates how ethnopharmacology is increasingly moving beyond descriptive accounts of traditional remedies toward mechanistic, evidence-based, and clinically relevant investigations. The collection was designed to critically assess contemporary developments in ethnopharmacology while identifying emerging opportunities associated with biodiversity conservation, sustainable development, artificial intelligence, systems pharmacology, and evidence-based validation of traditional medicine. The collection emphasizes scientific rigor, sustainability, translational relevance, and interdisciplinary collaboration, which are principles now central to the future of natural product research and nature-based drug discovery.

A considerable strength of this special issue is its breadth. The mass number of contributions collectively explore medicinal plants, traditional formulations, phytochemicals, and bioactive natural products across several therapeutic domains, including cancer, cardiovascular disease, inflammatory disorders, neurodegeneration, metabolic syndromes, kidney disease, dermatology, gastrointestinal disorders, and regenerative medicine. There are multiple reviews included within the special issue that focus on the molecular mechanisms underlying the pharmacological activity of traditional medicines, specifically the modulation of inflammatory pathways, oxidative stress, immune signaling, gut microbiota interactions, apoptosis, and metabolic regulation. Furthermore, many of the review articles emphasize the importance of multi-target pharmacological mechanisms, particularly in the context of complex herbal medicines where synergistic interactions may underlie therapeutic efficacy. For example, Huang et al. add value to this theme by elucidating the multi-target mechanisms of *Eucommia ulmoides* polysaccharides-based traditional herbal interventions in metabolic and inflammatory disease contexts, reinforcing the role of gut–liver and immune regulatory axes in ethnopharmacological efficacy. The findings support the systems-level view that complex formulations rather act through coordinated modulation of metabolic homeostasis than single-pathway targeting.

A particularly prominent theme throughout the collection is Traditional Chinese Medicine (TCM). It reflects the expanding integration of classical herbal systems with contemporary biomedical approaches. For example, Lu et al. present a review of the therapeutic potential of *Astragalus membranaceus* in atopic dermatitis and emphasize its modulation of the gut–skin axis and inflammatory pathways. Zhou et al. examine the bioactive metabolites and therapeutic applications of *Gastrodia elata*, while Wang et al. analyze recent advances in *Bletilla striata* polysaccharides and include assessments of the extraction methods, pharmacological mechanisms, and drug-delivery applications. Liu et al. further explored herbal formulas that specifically target macrophage polarization in atherosclerosis to showcase escalating interests in immunomodulatory mechanisms within ethnopharmacology. Wu et al. provide a systematic review demonstrating that traditional Chinese medicine may alleviate chemotherapy-induced diarrhea through multi-target modulation of intestinal inflammation and epithelial barrier function. These studies are just a few examples that reinforce the concept that multi-component herbal medicines may exercise therapeutic effects through multi-target mechanisms rather than single-molecule activity.

Additionally, the special issue includes broader phytopharmacological perspectives to address chronic and degenerative diseases. Ouyang et al. review natural bioactive compounds that are involved in bone metabolism and regenerative medicine. Liu & Li. discuss the therapeutic potential of phytoestrogens that occur in traditional medicine preparations for treatment of chronic kidney disease. Ma et al. explore how multi-component Chinese medicines may alleviate chronic cerebral hypoperfusion injury through integrated molecular mechanisms. Collectively, these studies illustrate how ethnopharmacology is increasingly contributing to systems-level understandings of disease modulation.

Moreover, the collection emphasizes an increasing awareness of biodiversity and regional medicinal traditions, showcasing impressive geographical and cultural diversity. Contributions examining Ayurveda, Latin American medicinal plants, African ethnomedicine, and Southeast Asian herbal traditions underscore the need to preserve indigenous knowledge systems, utilizing contemporary scientific methodologies to validate their therapeutic potential. Núñez-Selles et al. provide a detailed ethnopharmacological review of the *Pinus* species–specifically the Hispaniolan pine (*Pinus occidentalis*)–and emphasize both its therapeutic potential and major research gaps. Their review provides an analysis reflecting how regional biodiversity and traditional knowledge can inform future drug discovery and conservation strategies. Such studies accentuate the magnitude that encouraging sustainable and equitable scientific utilization have on preserving indigenous knowledge systems and regional medicinal biodiversity.

Cui et al. consolidate the existing knowledge on *Staphylea arguta* but also propose a foundation for its quality standardization, while Zhang et al. reviewed the botany, artificial cultivation, phytochemistry, ethnopharmacology, pharmacology, toxicology, and comprehensive utilization of *Aster tataricus*. Bai et al. focus on the botany, ethnopharmacology, phytochemistry, pharmacological effects, and clinical applications of *Carthamus tinctorius*. Moges et al. explore the phytochemical profiles and associated biological activities of *Myrsine africana* and Yang et al. provide an ethnobotanical, phytochemical, pharmacological analysis, with a focus on hepatotoxicity of *Psoralea corylifolia*. Special attention is directed towards flavonoids, recognized as a key metabolite group possessing broad utility in managing atopic dermatitis (Li et al.) and exhibiting anticancer effects (Dubey et al.). Other metabolites that are explored in the article collection are Astragaloside IV for its potential in the treatment of gastric cancer (Hu et al.), paeoniflorin for its potential in the treatment of depression (Hou et al.), berberin for its numerous pharmacological properties (Fan et al.), and quercetin for its potential applications in the treatment of acne vulgaris (Bo and Li).

The collection clearly demonstrates the increasing methodological sophistication of the field of ethnopharmacology. It presents multiple reviews analyzing the use of network pharmacology, metabolomics, systems biology, bioinformatics, and AI-assisted analytical approaches to decipher the complexity of herbal medicines. At the same time, the special issue repeatedly stresses the importance of rigorous phytochemical profiling and critical assessment of experimental design, while also acknowledging challenges associated with reproducibility, standardization, phytochemical characterization, and clinical validation. These are all issues that remain central to the credibility and translational success of ethnopharmacology. Furthermore, the editorial framework explicitly encourages authors to follow best-practice ethnopharmacological guidelines and the ConPhyMP standards for medicinal plant research (e.g., (Heinrich et al.)).

Importantly, the collection places strong emphasis on sustainability and ethical responsibility. Across these contributions, authors recognize that biodiversity conservation, equitable access to traditional knowledge, and community engagement are inseparable from the advancement of ethnopharmacology, now and in the future. By explicitly aligning with themes connected to the United Nations Sustainable Development Goals (SDGs), the special issue positions ethnopharmacology not merely as a biomedical discipline, but as a field profoundly intertwined with environmental stewardship, cultural preservation, and global health equity. This framing supports the notion that ethnopharmacology is not solely concerned with drug discovery, but rather with protecting traditional knowledge systems and promoting sustainable healthcare models.

In conclusion, the impact of the Research Topic “Bridging Traditional Knowledge and Modern Biomedical Innovation (Editorial: Reviews in Ethnopharmacology: 2025)” is fairly evident through its broad international participation and substantial readership. With hundreds of authors contributing and more than two hundred thousand cumulative views and downloads reported across the collection, the issue reflects the growing scientific and public interest in evidence-based traditional medicine research.

Collectively, the 48 publications featured in *Reviews in Ethnopharmacology: 2025* provide a comprehensive overview of current directions in the field while identifying important gaps for future inquiry. The issue successfully demonstrates that ethnopharmacology is no longer confined to documenting traditional remedies but has rather become a dynamic and integrative scientific discipline capable of contributing meaningfully to modern pharmacology, translational medicine, and global public health. By bridging ancient healing traditions with modern pharmacological science, the special issue contributes meaningfully to ongoing efforts to develop safer, more sustainable, and culturally informed therapeutic strategies for the future. As ethnopharmacology continues to evolve, this special issue stands as both a reflection of current achievements and a roadmap for future innovation.

